# Approaches to Identifying health disparities by ethnicity using linked Australian Census data

**DOI:** 10.23889/ijpds.v9i5.2507

**Published:** 2024-09-18

**Authors:** Fiona Stanaway, Liz Allen, Lin Zhu, Bree McDonald, Mei Ling Yap, Michelle Dickson, Natasha Nassar

**Affiliations:** 1 University of Sydney; 2 Australian National University; 3 The George Institute for Global Health, UNSW; 4 South Western Sydney Local Health District

## Background

Ethnicity is not collected routinely in Australian health data. Ancestry data is collected in the Census which is now linked to health data. We aimed to develop an approach to using ancestry data to examine health disparities.

## Methods

An expert panel was formed with representatives from the Federation of Ethnic Communities’ Councils, Australian Bureau of Statistics (ABS) and researchers. The work was also informed by a community panel and yarning circles led by Aboriginal and Torres Strait Islander academics. We analysed 2016 Census data linked to death registrations for 2016-2021 in 20.9 million people and compared how different approaches to categorising ethnicity impacted on detection of health inequalities between groups.

## Results

We developed the following approach: (1) Recategorise those reporting Australian only or New Zealand only ancestry from the Oceania to the European category; (2) For those reporting two ancestries: prioritise ethnic minority over national identities (e.g. Australian) and for the remainder create multiethnic groups based on the combined ancestries; (3) First analyse data at the maximum level of granularity, but then collapse up granularity where no disparities are identified to simplify results and messaging. Analysis of mortality data demonstrated that these approaches improved identification of ethnic mortality inequalities.

## Discussion

We developed a set of principles to use Australian Census Ancestry data to identify health disparities by ethnicity. The recommendations facilitate harnessing valuable Census data to identify important health disparities and better inform future health policy in Australia.

